# Comprehensive transcriptome analysis reveals genes potentially involved in isoflavone biosynthesis in *Pueraria thomsonii* Benth

**DOI:** 10.1371/journal.pone.0217593

**Published:** 2019-06-04

**Authors:** Meijun He, Yiwei Yao, Yanni Li, Meng Yang, Yu Li, Bin Wu, Dazhao Yu

**Affiliations:** 1 College of Life Science, Wuhan University, Wuhan, China; 2 Institute of Chinese Medicinal Materials, Hubei Academy of Agricultural Sciences, Enshi, China; 3 Center of Bioinformatics, Institute of Medicinal Plant Development, Chinese Academy of Medical Sciences and Peking Union Medical College, Beijing, China; Universidad Politecnica de Cartagena, SPAIN

## Abstract

*Pueraria thomsonii* Benth is an important medicinal plant. Transcriptome sequencing, unigene assembly, the annotation of transcripts and the study of gene expression profiles play vital roles in gene function research. However, the full-length transcriptome of *P*. *thomsonii* remains unknown. Here, we obtained 44,339 nonredundant transcripts of *P*. *thomsonii* by using the PacBio RS II Isoform and Illumina sequencing platforms, of which 43,195 were annotated genes. Compared with the expression levels in the plant roots, those of transcripts with a |fold change| ≥ 4 and FDR < 0.01 in the leaves or stems were assigned as differentially expressed transcripts (DETs). In total, we found 9,225 DETs, 32 of which came from structural genes that were potentially involved in isoflavone biosynthesis. The expression profiles of 8 structural genes from the RNA-Seq data were validated by qRT-PCR. We identified 437 transcription factors (TFs) that were positively or negatively correlated with at least 1 of the structural genes involved in isoflavone biosynthesis using Pearson correlation coefficients (r) (r > 0.8 or r < -0.8). We also identified a total of 32 microRNAs (miRNAs), which targeted 805 transcripts. These miRNAs caused enriched function in ‘ATP binding’, ‘defense response’, ‘ADP binding’, and ‘signal transduction’. Interestingly, *MIR156a* potentially promoted isoflavone biosynthesis by repressing *SBP*, and *MIR319* promoted isoflavone biosynthesis by repressing *TCP* and *HB-HD-ZIP*. Finally, we identified 2,690 alternative splicing events, including that of the structural genes of trans-cinnamate 4-monooxygenase and pullulanase, which are potentially involved in the biosynthesis of isoflavone and starch, respectively, and of three TFs potentially involved in isoflavone biosynthesis. Together, these results provide us with comprehensive insight into the gene expression and regulation of *P*. *thomsonii*.

## Introduction

*Pueraria thomsonii* Benth belongs to the perennial leguminous plants, and it is an important Chinese medicinal herb that is mainly distributed in East Asian countries, such as China and Japan. The dried roots of *P*. *thomsonii* were first described in the *Shengnong Bencao Jing* as a pungent diaphoretic drug, which could be used to treat influenza, body stiffness, and other illnesses [1]. The main active isoflavones in *P*. *thomsonii* are puerarin and daidzein, which accumulates in roots for *Pueraria* plants [1–5]. Modern pharmacological research has shown that puerarin can be used to treat diabetes and vascular hypertension [6], as well as to promote osteogenic differentiation and bone formation [7]. Daidzein can be used to decrease blood alcohol levels [8]. The biosynthesis of isoflavone is via phenylpropanoid and isoflavonoid hybrid-pathway [3–6,9,10]. For the phenylpropanoid pathway, starting from phenylalanine, liquiritigenin and naringenin are formed through the successive catalysis of phenylalanine ammonia-lyase (PAL), trans-cinnamate4-monooxygenase (CA4H) and 4-Coumarone Coenzyme A Ligase (4CL), 6-deoxychalcone synthase (CHS) and chalcone isomerase (CHI). The isoflavonoids biosynthetic pathway has been studied intensively in *Pueraria lobata* (Willd.) Ohwi, another important *Pueraria* plant. Firstly, liquiritigenin or naringenin are hydroxylated by 2-hydroxyisoflavanone synthase (IFS) and 2-hydroxyisoflavone dehydrate (HID) to form daidzein, genistein and formononetin. Then these isoflavone scaffolds are further modified by glycosyltransferases and methyltransferases, yielding to diverse isoflavonoids [3–5,10]. In addition, the roots of *P*. *thomsonii* are rich in starch and have a high pasting temperature, gelatinization temperature and enthalpy [11].

However, current research on the molecular biology of *P*. *thomsonii* is limited. EST information is not available, and only 46 nucleotide sequences have been deposited in the NCBI database. Additionally, there have not been any studies on protein-coding candidate genes involved in isoflavone biosynthesis in *P*. *thomsonii*, and microRNAs (miRNAs) in *P*. *thomsonii* have not been reported. miRNAs are a class of 20–24 nt noncoding RNAs that play vital roles in plant morphogenesis, biotic and abiotic stresses, and the synthesis of secondary metabolites through either cleavage of their targets or by repressing target translation at the posttranscriptional level [12,13].

For species without genome information, high-throughput RNA sequencing and data analysis are powerful tools to mine for genes that have specific functions. Compared to the short-read Illumina sequencing platform, PacBio Iso-Seq can obtain long reads of up to 10 kb and does not require reconstruction of the transcripts, making it convenient and accurate to find splice variants [14]. The full-length transcriptome has been previously described for some other medicinal plants, such as *Salvia miltiorrhiza* [14] *Dendrobium officinale* [15], *Astragalus membranaceus* [16] and *Cassia obtusifolia* [17].

In this study, we used PacBio RS II and illumina sequencing to obtain the full-length transcripts from *P*. *thomsonii* and carry out comprehensive analysis. In addition to the structural genes, we found regulatory genes, such as transcription factors (TFs) and miRNAs were potentially involved in isoflavone biosynthesis. We also identified alternative splicing (AS) events, some of which were related to the biosynthesis of isoflavone and starch. These results lay the foundation for breeding *P*. *thomsonii* cultivars with high isoflavone compounds through engineering.

## Materials and methods

### Plant materials and RNA extraction

Two-year-old “Enge-8” plants were grown in Huazhong Medicinal Botanical Garden of Institute of Chinese Medicinal Materials, Hubei Academy of Agricultural Sciences (Enshi, China). At that time, the temperature ranged from 19°C to 32°C, averaging approximately 25°C. Leaves, stems and roots were collected from 3 plants at an early stage on July 12, 2017, and frozen in liquid nitrogen until further use. Total RNA was extracted using RNAprep Pure Plant Kit (DP441) (TIANGEN Biotech Co., Ltd) according to the manufacturer's instructions. RNA degradation and contamination were assessed on 1.5% agarose gels. Then, the total RNA for the construction of the RNA sequencing libraries was quantified with a NanoDrop (Thermo Scientific), and the RNA integrity was assessed with the Agilent Technologies 2100 Bioanalyzer system (Santa Clara, CA) with a cutoff of an RNA integrity number (RIN) greater than 7.

### PacBio Iso-Seq library preparation and sequencing

Equal quantities of total RNA from the nine individual samples described above were pooled for PacBio Iso-Seq library construction. Poly (A) RNA was isolated from the total RNA using oligo(dT) magnetic beads and the Poly (A) PuristTM Kit. Isolated poly (A) RNA was eluted with 20 μl of RNase-free water. One microgram of RNA was reverse transcribed using the Clontech SMARTer cDNA synthesis kit. After PCR amplification, quality control, and purification, we performed size selection using the BluePippin Size Selection System protocol, which produced two fractions containing fragments of 1–3 kb and 3–6 kb in length. These cDNA products were used to construct libraries using the SMRTBell Template Prep Kit. The concentration and quality of the cDNA library were measured using the Qubit 2.0 fluorometer and Agilent 2100 Bioanalyzer, respectively. Finally, a total of four SMRT cells were sequenced on the PacBio RS II platform (Pacific Biosciences, Menlo Park, CA, USA).

### Illumina transcriptome library construction and sequencing

The Poly (A) mRNA from 9 samples (three organs from three plants) was enriched from the total RNA using oligo (dT) magnetic beads. Following the enrichment, the mRNA was fragmented into small pieces. Using these short fragments as templates, the first-strand cDNA was synthesized using Superscript III reverse transcriptase and random hexamer (N6) primers. Subsequently, the RNA templates were removed, and the second-strand cDNA was synthesized using dNTPs, DNA polymerase I, and RNase H. These short double cDNA fragments were purified with AMPure XP beads. After end reparation and A-tailing, the short cDNA fragments were ligated with the Illumina paired-end adaptors and purified with AMPure XP beads. Then, PCR was used to selectively enrich DNA fragments with adapter molecules on both ends and to create the final cDNA library. The concentration of the cDNA library was assessed using the Qubit2.0 fluorometer (Life Technologies, Carlsbad, CA, USA), and the quality of the cDNA library was measured using the Agilent 2100 Bioanalyzer (Santa Clara, CA). Finally, the libraries were sequenced from both the 5’ and 3’ ends using the Illumina sequencing system (Illumina, San Diego, CA, USA).

### PacBio data analysis

SMRT Analysis software package (v2.3.0) was used for data processing. First, raw reads were error corrected and processed into reads of insert (ROIs) using the ToFu pipeline [18]. Next, full-length, nonchimeric (FLNC) transcripts were identified by searching for the polyA tail signal and the 5’ and 3’ cDNA primers in ROIs. ICE (Iterative Clustering for Error Correction) was used to obtain consensus isoforms, and FL consensus sequences from ICE were polished using Quiver [19]. FL transcripts with postcorrection accuracy above 99% were generated for further analysis. All the isoforms were corrected with the help of the Illumina short reads using the Proovread tool [20] with default settings. Finally, the longest isoform from each cluster was regarded as the transcript to be followed up on with correction by CD-HIT software (v4.6) [21] with the following parameters: -c 0.99 –G 0 –aL 0.00 –aS 0.99 –AS 30 -M 0 –d 0 –p 1 to remove all redundant transcripts.

### Detection of AS events

The AS events were obtained as previously described [16]. First, the nonredundant transcripts were processed with the Coding GENome reconstruction Tool (Cogent v1.4, https://github.com/Magdoll/Cogent). In general, Cogent first creates the k-mer profile of nonredundant transcripts, calculates pairwise distances, and then clusters transcripts into families based on their k-mer similarity. Each transcript family is further reconstructed into one or several unique transcript model(s) (UniTransModels) using a De Bruijn graph method. Then, nonredundant transcripts were mapped to UniTransModels using GMAP (v2014-08-04) [22]. Splicing junctions for transcripts that mapped to the same UniTransModels were examined, and transcripts with the same splicing junctions were collapsed. Collapsed transcripts with different splicing junctions were identified as transcription isoforms. AS events were detected with SUPPA using the default settings [23].

### Gene functional annotation

Nonredundant transcripts were annotated based on the following databases: Nr (NCBI nonredundant protein sequences) (https://blast.ncbi.nlm.nih.gov/Blast.cgi), Swissprot [24], KEGG (Kyoto Encyclopedia of Genes and Genomes) [25], GO (Gene Ontology) [26], Clusters of Orthologous Groups of proteins (COG) [27], evolutionary genealogy of genes: Non-supervised Orthologous Groups (eggNOG) [28], euKaryotic Ortholog Groups (KOG) [29] and Pfam [30] using E-value 10–5 as a cutoff.

### Illumina data processing and Quantification of gene expression levels

Raw reads in FASTQ format were first processed using in-house Perl scripts. Clean reads were obtained by removing reads containing adapters, reads containing poly-N and low-quality reads. Gene expression levels were determined by calculating the sum of the fragments mapping to each transcript and were then normalized by converting the fragment counts to fragments per kilobase of transcript per million mapped reads, or FPKM, and were then normalized to the nonredundant transcripts. Differential expression analysis was performed using DESeq (v 1.10.1) [31]. Genes that had a |fold change| ≥ 4 and FDR < 0.01 found by DESeq were assigned as differentially expressed.

### MiRNA identification and target prediction

Known microRNA sequences were downloaded from miRBase (Released 22, http://www.mirbase.org) [32]. Due to the conservation of miRNAs, the known sequences of the miRNAs in the miRBase database were compared with the nonredundant transcript data with the use of predicted precursor module “psRobot-mir” of the psRobot software [33]. The criteria described by Meyers et al were applied to check *P*. *thomsonii* microRNAs manually [34,35]. When two or more miRNAs were located at the same precursor, the sequences of the *Glycine max* or *Medicago truncatula* miRNA were chosen as a reference. The minimal folding free energy index (MFEI) was calculated as described previously [36]. Targets of the microRNAs were predicted by the psRNATarget Server using a penalty score ≤ 3 [37].

### Validation the expression profile of transcripts by qRT-PCR

For each tissue type, the RNA samples extracted from three individuals were used as biological replicates for qRT-PCR analyses. Each biological replicate had three technical replicates. Then, RNA was digested with RNase-free DNase I (Promega) to remove genomic DNA contamination. Reverse transcription was performed using 1 μg total RNA for each example with 200 U M-MLV Transcriptase (Takara) in a 20 μl volume. The reaction was carried out at 70°C for 10 min, 37°C for 60 min and 70°C for 15 min. The resulting cDNA was diluted to 800 μl with sterile water. qPCR was carried out in triplicate reactions using the BIO-RAD CFX system (BIO-RAD). Gene-specific primers were designed by Primer3 (http://bioinfo.ut.ee/primer3/). The primers used in this study are listed in S1 Table. A 40S ribosomal protein S8 (PB8162) was chosen as an endogenous control because it was relatively stable in our RNA-Seq experiments and has been described previously [38]. PCR was carried out in a 15 μl volume containing 3 μl diluted cDNA, 250 nM forward primer, 250 nM reverse primer, and 1×SYBR Premix Ex Taq II (TaKaRa) using the following conditions: 95°C for 3 min, followed by 40 cycles of 95°C for 15 sec, 59°C for 15 sec and 72°C for 15 sec. Melting curve analyses were performed to verify the specificity with the Bio-Rad CFX Manage software. Relative expression levels were calculated using the 2^-ΔΔCt^ method [39]. The Pearson correlation coefficient of the expression profiles was also calculated.

## Results

### Combined sequencing of *P*. *thomsonii* transcripts

To deeply mine the gene information of *P*. *thomsonii*, sequencing with the PacBio RS II and Illumina platforms was performed simultaneously. First, full-length cDNA libraries from the pooled poly (A) RNA samples were constructed and subjected to SMRT sequencing by the PacBio RS II platform. To avoid the preference of different transcripts based on sequence length, 1–3 kb and 3–6 kb libraries were constructed and sequenced by 2 SMRT cells. In total, 300,584 polymerase reads were generated for these two libraries. After discarding the adapter sequence and sequences shorter than 50 bp, 207,253 and 237,356 subreads representing 5.13 Gb and 5.88 Gb were obtained, respectively (S2 Table). Next, 160,327 and 147,869 ROIs with high quality were extracted with a mean length of 1,646 bp and 3,270 bp, respectively (S3 Table). Next, we further distinguished the full-length nonchimeric sequences from the non-full-length sequences (S4 Table). Based on the clustering algorithm of ICE, we integrated the 1–3 kb and 3–6 kb libraries and found 76,347 consensus isoforms with a mean length of 2,251 bp. These data contained 62,400 high-quality transcripts and 13,947 low-quality transcripts (Table 1).

**Table 1:** Results of ICE analysis.

cDNA Size	Number of consensus isoforms	Average consensus isoform read length	Number of polished, high-quality isoforms	Number of polished, low-quality isoforms	Percent of polished, high-quality isoforms (%)
0–1 kb	12,669	815	11,805	864	93.18%
1–2 kb	26,790	1,418	23,546	3,244	87.89%
2–3 kb	10,328	2,599	8,320	2,008	80.56%
3–6 kb	25,965	3,519	18,651	7,314	71.83%
> 6 kb	595	8,991	78	517	13.11%
All	76,347	2,251	62,400	13,947	81.73%

cDNA size: insert fragment size of cDNA; Number of consensus isoforms: the number of consensus isoforms obtained from ICE clustering analysis; Average consensus isoform length: sequence length of consensus isoform; Number of HQ isoforms: the number of high-quality transcripts; Number of LQ isoforms: the number of low-quality transcripts; Percent of HQ isoforms (%): percentage of high-quality transcripts in consensus isoform.

All the 76,347 consensus isoforms were further corrected using the Illumina read data (S5 Table) to improve quality. Finally, after removing the redundant sequences and the cluster of the low-quality transcripts using CD-HIT (c = 0.99), a total of 44,339 nonredundant transcripts were obtained, which were further annotated for downstream analysis.

For Illumina sequencing, 9 mRNA samples from the leaves, stems and roots (each in triplicate) of P. thomsonii were subjected to sequencing by Illumina HiSeq. As a result, we obtained 23,147,850, 23,885,655, 22,604,617, 40,145,343, 41,575,513, 34,787,110, 25,765,267, 44,547,852 and 27,659,075 clean reads, which is representative of 848.9 Gb (S5 Table). The Illumina sequencing reads were not assembled alone because approximately 80% of them mapped to the 44,339 nonredundant transcripts (S6 Table).

### Gene function annotation and categorization

**Fig 1:**
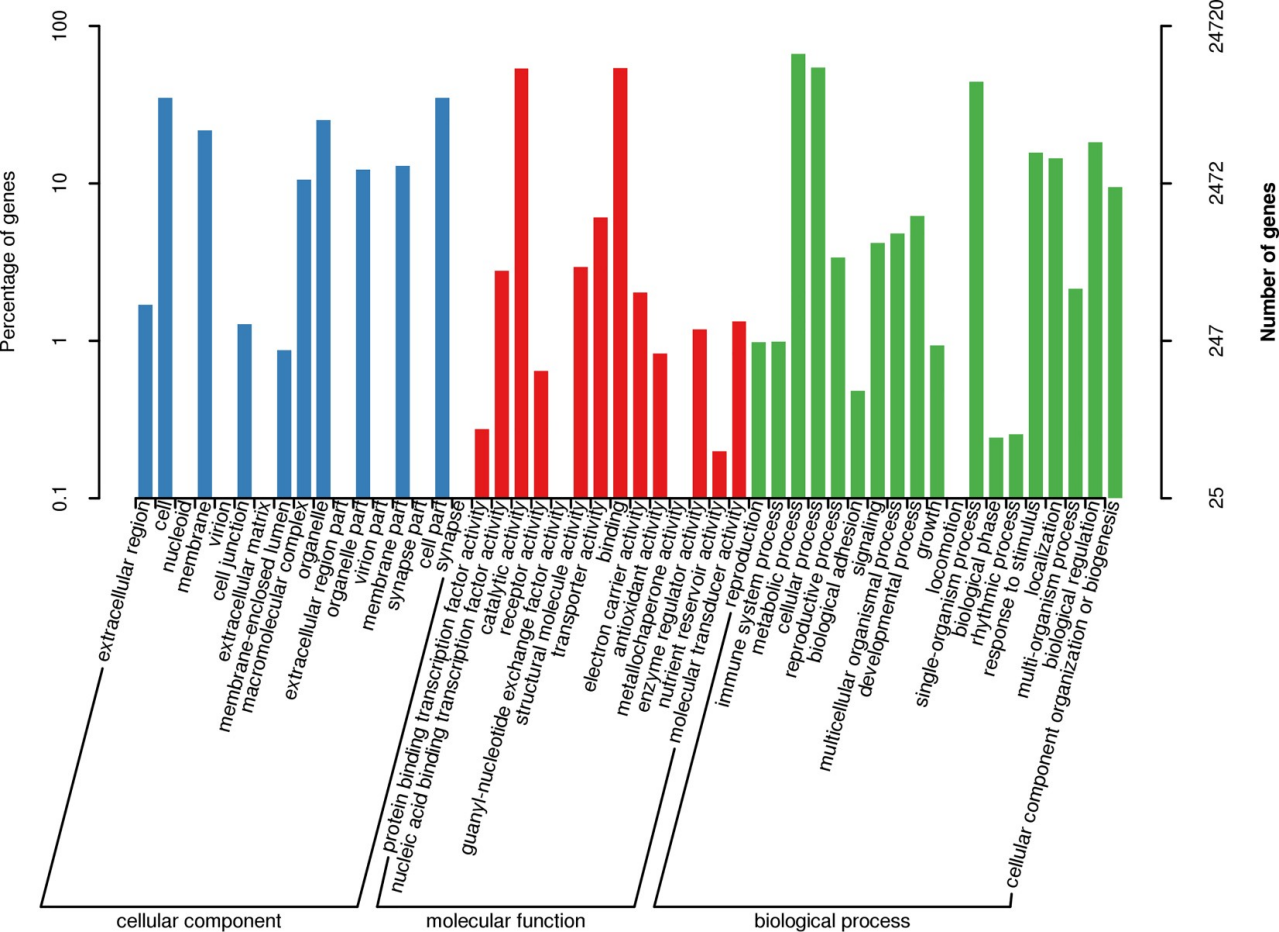
GO annotation of *P*. *thomsonii* transcripts. Percent of transcripts assigned to partial subcategories of the cellular component (blue), molecular function (red) and biological process (green) categories are shown.

To categorize the nonredundant transcripts, sequences were compared against Nr, Swissprot, KEGG, GO, COG, eggNOG, KOG and Pfam using BLASTX; as a result, 43,083, 32,079, 17,391, 24,720, 40,870, 25,626 and 35,525 transcripts were found to be annotated in the abovementioned databases, respectively. These results are summarized in S7 Table, and a total of 43,195 transcripts were annotated. The databases COG, eggNOG, KOG and Pfam distinguish proteins by different domains/families. GO terms describe the functions of these cross-species homologous genes and their gene products, which are further divided into biological processes, cellular locations and molecular functions. In this study, 45,138, 17,048 and 37,654 GO terms were obtained for the three categories. Fig 1 shows the GO assignments of 40 subcategories within the three categories, in which metabolic process (GO:0008152), catalytic activity (GO:0003824), binding (GO:0005488), cellular process (GO:0009987), and single-organism process (GO:0044699) ranked in the top five.

### Identification of structural genes involved in isoflavone and starch biosynthesis

Isoflavones are the main active compounds of *P*. *thomsonii*. However, isoflavone-related genes in *P*. *thomsonii* have not been reported to date. To fill this gap, we first annotated the 44,339 nonredundant transcripts of *P*. *thomsonii* with KEGG database. In total, 17,391 transcripts were annotated, which could be divided into 128 categories (S8 Table). Among them, carbohydrate metabolism (ko01200), biosynthesis of amino acids (ko01230), ribosome (ko03010), protein processing in endoplasmic reticulum (ko04141) and spliceosome (ko03040) ranked in the top five. In total, 4,134 and 2,072 transcripts were involved in metabolic pathways and the biosynthesis of secondary metabolites, respectively. Based on KEGG and Nr annotation, we annotated a total of 69 transcripts that could be associated with the isoflavone biosynthesis (S1 Fig, S9 Table). Compared with *P*.*lobata*, some genes were absent in *P*. *thomsoni*i, such as 3'-O-methyltransferase, isoflavone-specific 4'-O-methyltransferase. These data greatly enrich our knowledge of isoflavone biosynthesis and provide candidate genes for genetic engineering to enhance the production of isoflavone compounds in *P*. *thomsonii*.

The roots of *P*. *thomsonii* are called Fen-Ge in Chinese because they are rich in starch [1]. Therefore, it is important to mine starch-related genes in *P*. *thomsonii*. Starch consists of amylose and amylopectin. Starch biosynthesis is a complex process that requires the coordinated activity of multiple enzymes. It is reported that amylose is mainly produced via granule-bound starch synthase (GBSS). Amylopectin synthesis involves more than 17 genes encoding different types of starch synthase (SS), starch branching enzyme (SBE), starch debranching enzyme (DBE) and phosphate transporter PHO1 (PHO1) [40]. In this study, we found that 10 families of genes were involved in starch synthesis (S10 Table), including *GBSS Ⅰa*, uncharacterized *GBSS*, *GBSS Ⅱ*, *SSⅢ*, *SSⅣ*, *SSSⅠ*, *SSSⅢ*, pullulanase-DBE (*PULⅠ*), *PHO1* and *PHO1*-like.

### Analysis of TFs

It has been reported that TFs play important roles in plant growth and development and in the biosynthesis of secondary metabolites [9]. Until recently, TFs have not previously been reported in *Pueraria* plants. To analyze TFs in *P*. *thomsonii*, sequences of the nonredundant transcripts were compared to the sequences in the iTAK database using the default parameters [41]. As a result, 2,079 TF transcripts were identified, which could be classified into 66 families (S11 Table). Among them, the bHLH, C3H, AP2/ERFERF, bZIP and C2H2 families of TFs ranked in the top five, followed by the MYB-related, B3-ARF, WRKY, FAR1, GRAS, and MYB TF families.

### Identification of DETs related to isoflavone biosynthesis

Then, the expression levels of the nonredundant transcripts were determined by FPKM. Compared with the expression levels in the plant roots, those of transcripts with a |fold change| ≥ 4 and FDR < 0.01 in the leaves or stems found by DESeq were assigned as DETs, because the roots accumulate more isoflavone compounds [1–5] and some structural genes related to isoflavone express higher in roots [3–6,10]. In total, 9,225 DETs were found (S12 Table). GO enrichment showed that the top 5 categories of DETs were genes associated with oxidation-reduction process (GO: 0055114), ATP binding (GO:0005524), integral component of membrane (GO:0016021), metal ion binding (GO:0046872) and membrane (GO:0016020) (S13 Table).

**Fig 2:**
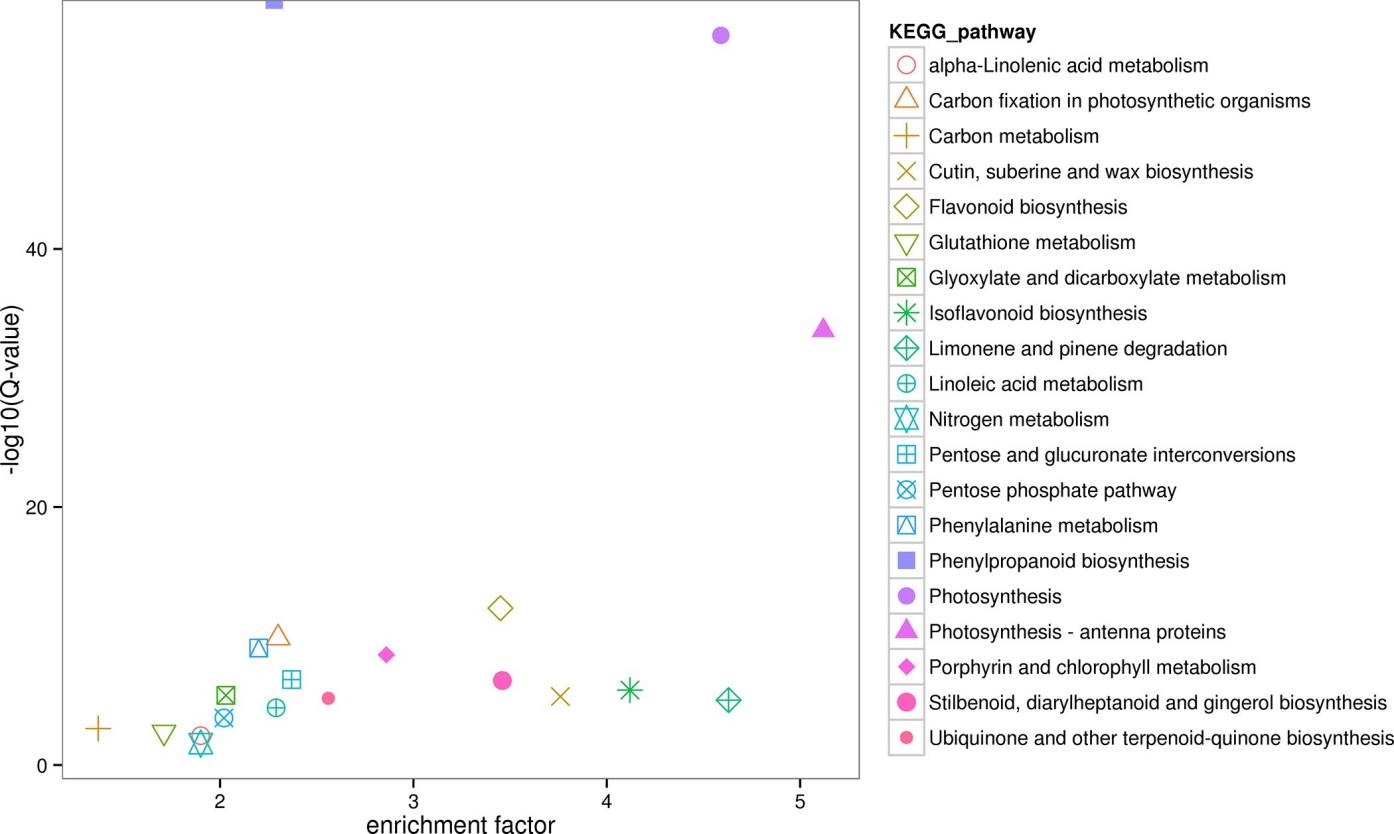
KEGG enrichment of DETs for the comparison of roots vs leaves. The X-axis represents the enrichment factor and log10 of the Q-value. The Y-axis indicates the different KEGG pathways.

Among the 9,225 DETs, the comparison of the roots and leaves resulted in 8,138 DETs. Of these, 4,610 were upregulated, and 3,528 were downregulated. KEGG enrichment was carried out, and the top 20 enriched pathways, including the phenylpropanoid- and isoflavone-biosynthesis pathways, are shown in Fig 2. A total of 4,381 DETs were found when the roots and stems were compared. Among these, 2,687 were upregulated, while 1,694 were downregulated. The phenylpropanoid- and isoflavone-biosynthesis pathways were also enriched (S2 Fig). Of the 9,225 total DETs, 36 were annotated as encoding structural biosynthesis genes (S8 Table), and 32 of these were potentially involved in isoflavone synthesis because they are more highly expressed in roots than in leaves or stems.

**Fig 3:**
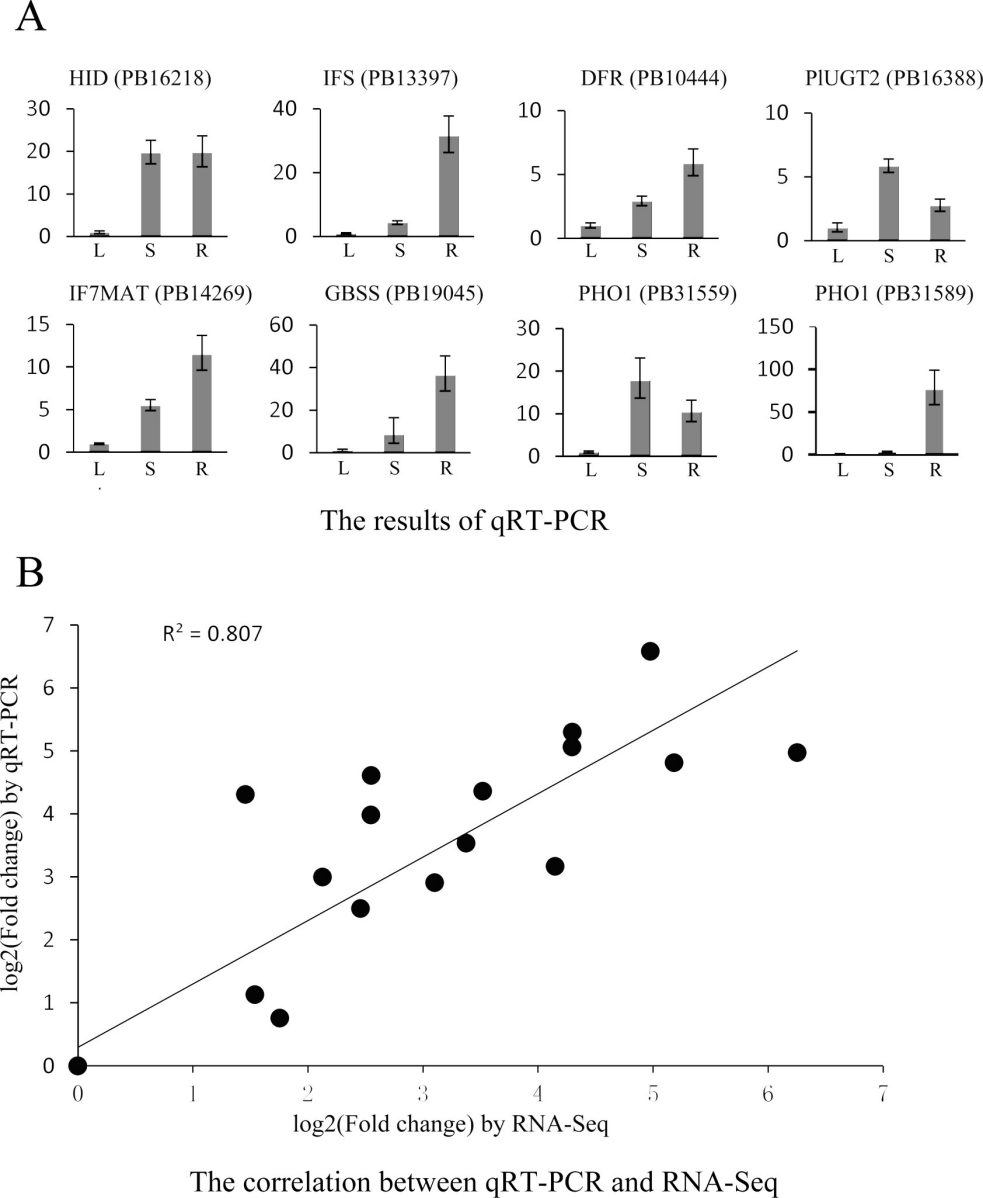
Expression profiles of 8 transcripts by qRT-PCR and the correlation of the results between qRT-PCR and RNA-Seq. Fold changes in the transcript levels of different tissues are shown in Fig 3A. The average expression levels in the plant leaves were set to 1. Error bars represent standard error. L, S and R represent the leaves, stems and roots, respectively. Fig 3B shows the correlation between the qRT-PCR and RNA-Seq expression levels. The X-axis represents log2-fold change in the expression levels found by qRT-PCR. The Y-axis indicates the log2 value of the expression level fold change from RNA-Seq.

To determine the reliability of DETs, we validated the RNA-Seq results (S14 Table) by quantitative reverse transcription-PCR (qRT-PCR). The expression profiles of 8 transcripts, including dihydroflavonol 4-reductase (*DRF*) (PB10444), *HID* (PB16218), *IFS* (PB13397), *PlUGT2* (PB16388), *IF7MAT* (PB14269), granule-bound starch synthase (PB19045) and *PHO1* (PB31559, PB31589), which are potentially involved in the biosynthesis of flavone, isoflavone or starch, respectively, were validated by qRT-PCR (Fig 3A). Pearson’s correlation coefficient was 0.807 (Fig 3B), indicating that the qRT-PCR results were positively related to the levels found by RNA-Seq.

In addition to the structural genes, the data were further analyzed to find other regulatory genes, such as TFs, that were potentially involved in the isoflavone biosynthesis. Among the 9,225 total DETs, 508 were predicted to be TFs. We hypothesized that isoflavone-related TFs should share highly correlated expression profiles with those of structural genes. Therefore, we calculated Pearson’s correlation coefficients (r) of the expression profiles of the 508 TFs with those of *CHS* (PB5670), *CHI* (PB36892), *HID* (PB16218), *IFS* (PB13397), *PlUGT2* (PB16388), *IF7MAT* (PB14269) and *PlUGT43* (PB16388). As a result, we found a total of 437 TFs that were coexpressed with at least one of the 7 structural genes involved in isoflavone biosynthesis using the cutoff r > 0.8 or r < -0.8 (S15 Table). Among the 437 TFs, 57, 12, 94, 173, 74, 20, and 7 were positively or negatively correlated with the 7, 6, 5, 4, 3, 2, and 1 structural gene, respectively (S15 Table). The 437 TFs could be classified into 47 families, and the MYB, HB-HD-ZIP, bHLH, C2H2, WRKY, and bZIP TF families were enriched. These data provided a foundation for further studies of the TFs involved in isoflavone biosynthesis.

### Predicted microRNAs and their targets

In order to decipher the regulatory role of miRNAs in isoflavone biosynthesis in *P*. *thomsonii*, we identified the miRNAs and analyzed their target genes. Because *P*. *thomsonii* small RNAs were not sequenced, based on the conservation of miRNAs, we used psRobot software to predict the *P*. *thomsonii* miRNAs by comparing the known miRNAs in miRBase (version 22) to the nonredundant transcripts to find matches with no more than three mismatches and less than two bulges in the hairpin structures. In total, we identified 32 miRNAs that belong to 12 families (Table 2).

**Table 2:** Identification of 32 *P*. *thomsonii* miRNAs.

miRNA name	miRNA sequence	Transcript ID (position)	MFEI*	Length (bp)
1507a-5p	GAGGUGUUUGGGAUGAGAGAA	PB8662|141|253|+	1.29	113
1507a-3p	CCUCAUUCCAAACAUCAUCU	PB8662|141|253|+	1.29	113
1507b-5p	GAGGUGUUUGGGAUGAGAGAA	PB3714|134|246|+	1.29	113
1507b-3p	CCUCAUUCCAAACAUCAUCU	PB3714|134|246|+	1.29	113
1507c	UCUCAUUCCAUACAUCGUCUGA	PB4622|448|590|+	0.97	143
156a	GACAGAAGAGAGUGAGCAC	PB8260|799|951|+	0.85	153
156b	UUGACAGAAGAGAGUGAGCAC	PB22484|800|952|+	0.85	153
156c-5p	CUGACAGAAGAGAGUGAGCA	PB23248|279|411|+	1.09	133
156c-3p	GCUCACUUCUCUUUCUGUCAGC	PB23248|279|411|+	1.09	133
159-5p	GAGCUCCUUGAAGUCCAAUU	PB17328|315|527|+	1.03	213
159-3p	UUUGGAUUGAAGGGAGCUCUA	PB17328|315|527|+	1.03	213
167a-5p	UGAAGCUGCCAGCAUGAUCU	PB18025|132|224|+	0.93	93
167a-3p	AGAUCAUGUGGCAGUUUCACC	PB18025|132|224|+	0.93	93
171a-5p	AGAUAUUGGUACGGUUCAAUC	PB25457|350|462|+	1.05	113
171a-3p	UGAGCCGUGCCAAUAUCACAU	PB25457|350|462|+	1.05	113
171b-5p	AGAUAUUGGUACGGUUCAAUC	PB33751|725|837|+	1.05	113
171b-3p	UGAGCCGUGCCAAUAUCACAU	PB33751|725|837|+	1.05	113
2089	UUACCUAUUCCACCAAUUCCAU	PB40844|2831|2963|+	1	133
2119a	UCAAAGGGAGUUGUAGGGGAA	PB673|316|478|+	1.78	163
2119b	UCAAAGGGAGUUGUAGGGGAA	PB273|367|529|+	1.75	163
2119c	UCAAAGGGAGUUGUAGGGGAA	PB3566|251|413|+	1.68	163
2119d	UCAAAGGGAGUUGUAGGGGAA	PB193|316|478|+	1.68	163
319-5p	AGAGCUUCCUUCAGUCCACUC	PB40981|1610|1822|+	1.08	213
319-3p	UUGGACUGAAGGGAGCUCCCU	PB40981|1610|1822|+	1.08	213
390-5p	UAAAGCUCAGGAGGGAUAGCG	PB932|102|234|+	0.89	133
390-3p	CGCUAUCCAUCCUGAGUUUCA	PB932|102|234|+	0.89	133
396a-5p	UUCCACAGCUUUCUUGAACU	PB39010|176|318|+	1.01	143
396a-3p	UUCAAUAAAGCUGUGGGAAG	PB39010|176|318|+	1.01	143
398a	UUGUGUUCUCAGGUCACCCCU	PB673|46|188|+	1.1	143
398b	UGUGUUCUCAGGUCACCCCUU	PB193|46|188|+	0.47	143
398c	UUGUGUUCUCAGGUCACCCCU	PB273|96|238|+	1.1	143
408a	UGCACUGCCUCUUCCCUGGCU	PB8254|383|535|+	0.82	153

* MFEI, minimal folding free energy index of the hairpin structures.

**Fig 4:**
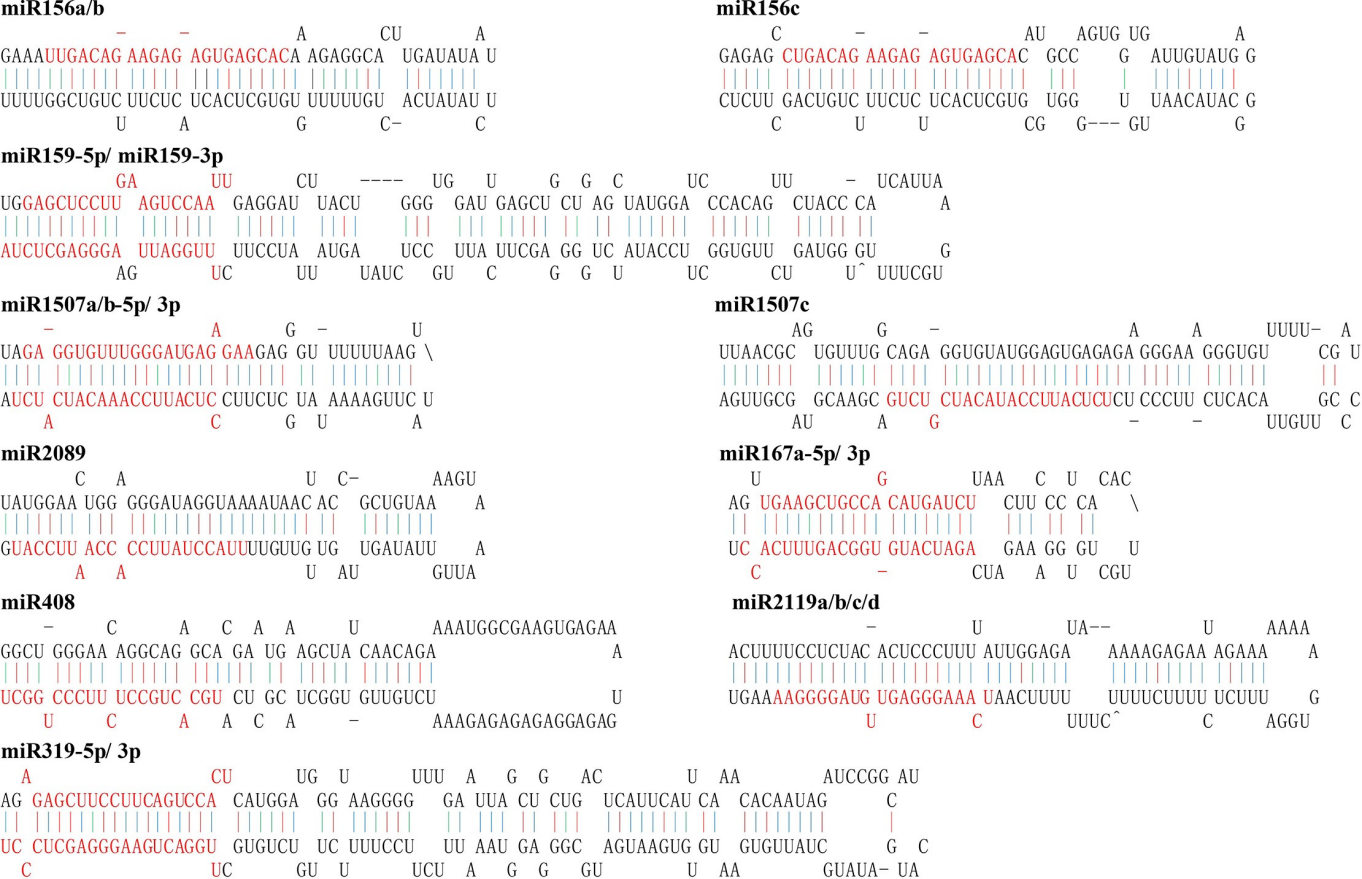
Predicted hairpin structures of *P*. *thomsonii* miRNA precursors. Mature miRNA sequences are indicated in red. The red, green and blue vertical lines indicate G:C, G:U and A:U pairing, respectively.

Among the identified miRNAs, *MIR156*, *MIR171*, *MIR398*, *MIR1507* and *MIR*2119 had two or three members, while *MIR167*, *MIR171*, *MIR319*, *MIR390*, *MIR396* and *MIR408* had only one member. Some hairpin structures of miRNA precursors are shown in Fig 4.

**Fig 5:**
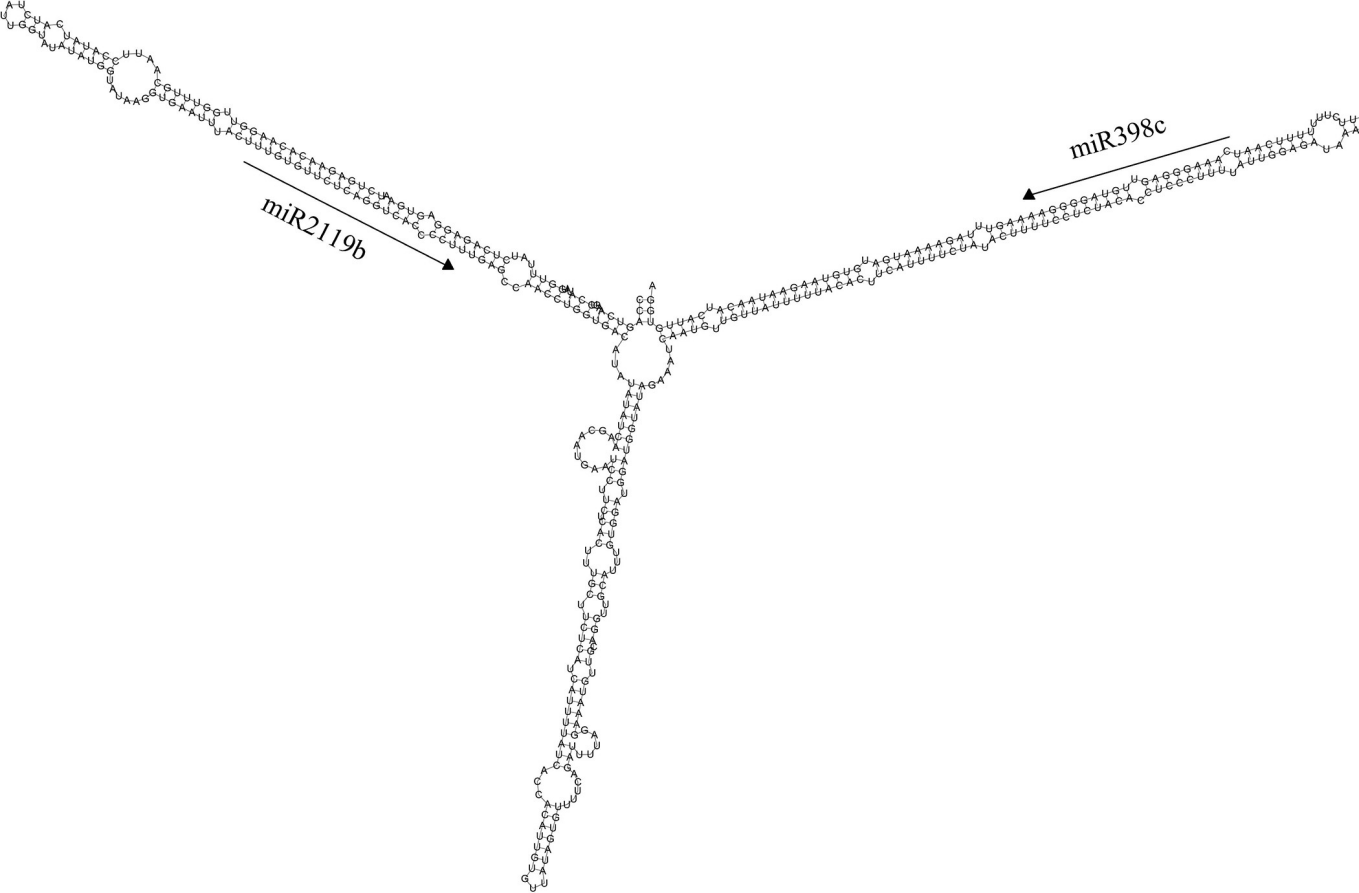
*MIR398c* and *MIR2119b* cluster at a single transcript.

Most miRNA precursors had MFEIs higher than 0.8, which was consistent with previous reports[36]. Interestingly, we found that the precursors of *MIR398c and MIR2119b* clustered at the PB273 transcript (Fig 5).

Moreover, these two miRNAs had no sequence similarity. In plants, clustering of miRNAs was frequently found in miRNAs from the same family or from different families but with sequence similarity [42].

The identified miRNAs targeted 805 transcripts (S16 Table), 608 of which were annotated. GO enrichment showed that the top 10 gene categories targeted by the identified miRNAs were those associated with ATP binding (GO:0005524), defense response (GO:0006952), ADP binding (GO:0043531), signal transduction (GO:0007165), integral component of membrane (GO:0016021), protein phosphorylation (GO:0006468), nucleus (GO:0005634), oxidation-reduction process (GO:0055114), protein serine/threonine kinase activity (GO:0004674) and zinc ion binding (GO:0008270). In total, we found 169 defense response targets. For example, *MIR2089* targeted 131 transcripts, of which 103 were TMV resistance protein N-like genes. For *MIR1507*, we predicted 163 targets, 47 of which were related to disease resistance. Moreover, we found 56 TF targets. Among them, *MIR159*, *MIR 167* and *MIR171* targeted the *MYB* (1 transcript), *LOB* (2 transcripts) and *GRAS* (2 transcripts) TFs, respectively. Some miRNAs targeted 2 families of TFs. For example, *MIR156* targeted *SBP* (16 transcripts) and *MYB* (1 transcript). *MIR1507* targeted *C3H* (3 transcripts) and *FAR1* (1 transcript). Some miRNAs targeted TFs from more than 2 families. For example, *MIR319* targeted *TCP* (5 transcripts), *C2C2-Dof* (2 transcripts) and *HB-HD-ZIP* (2 transcripts), while *MIR396* targeted *GRF* (3 transcripts), *HB-WOX* (3 transcripts), *RWP-RK* (1 transcript), *WRKY* (1 transcript) and *Trihelix* (1 transcript). Interestingly, we found that *SBP* (PB22265, PB8085) was targeted by *MIR156a*. The expression profile of PB22265 was negatively correlated with that of *IFS*, and PB8085 was negatively correlated with *CHS* and *IFS* (S15 Table). Therefore, we inferred that *MIR156a* promotes isoflavone biosynthesis by repressing *SBP* (PB22265, PB8085). We also found that *TCP* (PB17199) and *HB-HD-ZIP (*PB19367) were targeted by *MIR319*. The expression profile of PB17199 was negatively correlated with that of *CHS*, *CHI*, *HID*, *IFS*, *PlUGT2*, *IF7MAT* and *PlUGT43*, and the expression profile of PB19367 was negatively correlated with that of *CHS*, *CHI*, and *IFS* (S15 Table). Therefore, we inferred that *MIR319* promotes isoflavone biosynthesis by repressing *TCP* and *HB-HD-ZIP*.

### AS identification

In order to mine the role of AS in the biosynthesis of isoflavone and starch, we identified and analyzed AS in *P*. *thomsonii*. Compared to assembling unigenes using the Illumina short reads by Trinity, PacBio Iso-Seq can detect AS from the nonredundant transcripts using a *de novo* alignment method, therefore identifying true AS events. We first constructed the full-length UniTransModels using Cogent (https://github.com/Magdoll/Cogent). Then, AS events were detected by aligning the nonredundant transcripts back to the UniTransModels with SUPPA using the default setting. In this study, we found a total of 1,482 UniTransModels occurrences of AS, involving 2,690 AS events (S17 Table). We found 1,558 instances of intron retention (IR), 143 of exon skipping (ES), 525 of alternative 3’ (A3), and 464 of alternative 5’ (A5). Among the 1,482 UniTransModels, 876 had only one AS instance, while in 606 UniTransModels, AS occurred more than once. We found 812 UniTransModels that could be assigned GO terms. GO enrichment showed that ATP binding (GO:0005524), integral component of membrane (GO:0016021), zinc ion binding (GO:0008270), nucleus (GO:0005634) and protein phosphorylation (GO:0006468) ranked in the top five categories of AS events, followed by oxidation-reduction process (GO:0055114), membrane (GO:0016020), protein serine/threonine kinase activity (GO:0004674), metabolic process (GO:0008152) and nucleotide binding (GO:0000166). Interestingly, we found that the structural genes of UniTransModel 8313_0|path1 encoding CA4H had IR AS occurrence, and UniTransModel 1409_0|path1encoding PUL had A3, A5 and IR AS events. CA4H and PUL are potentially involved in the biosynthesis of isoflavone and starch, respectively. Three UniTransModels encoding TFs that are potentially involved in isoflavone biosynthesis had AS events (S15 and S17 Tables). 5170_2|path7 encoding WRKY incurred IR, while 8167_0|path0 encoding MYB59-like protein had both A5 and IR AS events. 1759_0|path1encoding HB also had an A5 AS occurrence.

## Discussion

In this study, we sequenced the full-length transcriptome of *P*. *thomsonii* for the first time and analyzed the gene expression profile. In total, we obtained 44,339 nonredundant transcripts, 43,195 of which were annotated. We found 9,225 DETs, and 32 of these were structural genes related to isoflavone *P*. *lobata* and *P*. *thomsonii* are the most important and widely used *Pueraria* plants. These two species were separated in 2005 because of differences in their isoflavone compounds. The roots of *P*. *lobata* and *P*. *thomsonii* are named Ge-Gen and Fen-Ge, respectively, in the Chinese Pharmacopoeia [1]. On the one hand, Ge-Gen contains higher levels of isoflavone compounds than Fen-Ge. Du et al found 14 major compounds in the roots of *P*. *lobata* and *P*. *thomsonii* by UHPLC-DAD-TOF-MS and found that the contents of puerarin ranged from 3.64–6.98 mg/g in *P*. *thomsonii* and 7.56–61.31 mg/g in *P*. *Lobata* [2]. Puerarin, mirificin and daidzin may serve as chemical markers to distinguish the two *Pueraria* species [2]. On the other hand, it was also reported that tectorigenin was primarily derived from the flowers of *P*. *thomsonii*, which had a potential beneficial role in Parkinson's disease via oxidative stress inhibition [43] and in Attenuates palmitate-induced endothelial insulin resistance[44]. Therefore, it is important to look for genes that may allow for breeding of *P*. *thomsonii* cultivars with high contents of special isoflavone compounds to extend its usage. In this study, we found structural genes that were potentially involved either upstream or downstream of the isoflavone pathway (Fig 3). One of these genes is *plUGT43*, which had been identified to function in C-glucosylation in *P*. *lobata* [5], indicating a common pathway in these two species. However, some structural genes in *P*. *lobata* were not found in *P*. *thomsonii*. One such gene is *3'-OMT*, which converts 3'-hydroxy-daidzein to 3'-methoxy-daidzein [3], indicating the existence of some individual pathways in the two species or different spatiotemporal gene expression.

Improving starch content is another breeding objective for *P*. *thomsonii* because starch is an important resource in food, feed and industrial material. In this study, we found 10 families of structural genes involved in starch synthesis. Because of the spatiotemporal expression specificity, some previously described starch-related genes [40] were absent from our transcriptome data. Future studies should integrate multiple ‘omics’ at different stages of tube development to reveal the mechanism of starch synthesis and to improve the yield of *P*. *thomsonii*.

In addition to the structural genes, we also studied regulatory genes, including TFs and miRNAs. TFs can regulate the expression levels of multiple flavonoid biosynthesis genes, which have drawn more attention in recent years. For example, in *Arabidopsis*, the expression levels of *PAL1*, *CHS* and *FLS* in *GmMYB12B2*-overexpressing plants were significantly higher than those in wild-type plants [45]. In soybean, *GmMYB29* promoted isoflavone biosynthesis by activating the *IFS2* and *CHS8* gene promoters [9]. *GmMYB39* inhibited isoflavone biosynthesis by repressing the transcript levels of *PAL*, *C4H*, *CHS*, *4CL* and *CHR* in soybean [46]. Here, we found that the expression profile of *MYB39* (PB15357) was negatively correlated with that of *CHS*, *CHI*, *IFS*, *IF7MAT* and *plUGT43* in *P*. *thomsonii*. In addition to MYBs, we also found TFs of WRKY, C2C2, HB, GRAS, bHLH, GARP and SBP that were potentially involved in isoflavone biosynthesis by activating or repressing multiple structural genes (S15 Table). In this study we only sequenced the transcriptome, which contains at most the 5’ untranslated regions of genes. Because the genome of *P*. *thomsonii* has not been released to date, we did not know the promoter sequences of interesting genes so that we could not analyze TF binding elements in their promoters region.

In this study, we also identified 33 miRNAs that targeted 805 transcripts involved in multiple biological processes. Of the 608 annotated targets, 169 were related to disease resistance, indicating the important role of *P*. *thomsonii* miRNAs in the defense response. Moreover, we found that *MIR156a* potentially promoted isoflavone biosynthesis by repressing *SBP*, while *MIR319* promoted isoflavone biosynthesis by repressing *TCP* and *HB-HD-ZIP*.

The identification of these structural genes and their associated regulatory genes could allow for the future gene engineering via synthetic biology to increase isoflavonoid content in *P*. *thomsonii*. Taken together, these results provide comprehensive insight into the gene expression and regulation of *P*. *thomsonii*.

## Supporting information

S1 Fig Proposed isoflavonoid biosynthetic pathway in *P. thomsonii*. PAL: Phenylalanine Ammonia-lyase; CA4H: Trans-cinnamate4-monooxygenase; 4CL: 4-Coumarone Coenzyme A Ligase; CHS: 6-deoxychalcone synthase; CHI: Chalcone isomerase; IFS: 2-hydroxyisoflavanone synthase; HID: 2-hydroxyisoflavone dehydrates; PLUGT43: Pueraria UDP glucosyltransferase 43; PLUGT2: Pueraria UDP glucosyltransferase 2; I7OMT: isoflavone-7-O-methyltransferase. IF7MAT: isoflavone 7-O-glucoside-6''-O-malonyltransferase.
(PNG)

S2 Fig KEGG enrichment of DETs for the comparison of roots vs stems. The X-axis represents the enrichment factor and log10 (Q-value). The Y-axis indicates the different KEGG pathways.
(PNG)

S1 Table Primers used for qRT-PCR. (DOCX)

S2 Table PacBio data analysis. (DOC)

S3 Table Reads of insert. (DOC)

S4 Table Statistics of full-length cDNA sequences. (DOCX)

S5 Table Statistics of Illumina-sequencing data. (DOC)

S6 Table Statistics of Illumina-sequencing mapping reads. (DOC)

S7 Table Functional annotation based on public databases. (DOC)

S8 Table Kegg category of transcripts. (XLS)

S9 Table Isoflavone-related genes in *P. thomsonii*. (XLS)

S10 Table Starch-related genes in *P. thomsonii*. (XLS)

S11 Table List of transcripts annotated as transcription factors. (XLS)

S12 Table Expression profiles of 9,225 DETs. (XLS)

S13 Table The top 40 GO assignments of 9,225 DETs. (XLS)

S14 Table The RNA-Seq results. (XLS)

S15 Table Expression profiles of 437 TFs were corrected with those of structural genes in isoflavone biosynthesis. (XLS)

S16 Table Predicted targets of miRNAs. (XLS)

S17 Table AS identification. (XLS)
