# Supplementary figures and images for: Comprehensive transcriptome analysis reveals genes potentially involved in isoflavone biosynthesis in Pueraria thomsonii Benth
Source: PLoS One. 2019 Jun 4;14(6):e0217593. doi: 10.1371/journal.pone.0217593 (PMC6548387; doi:10.1371/journal.pone.0217593)

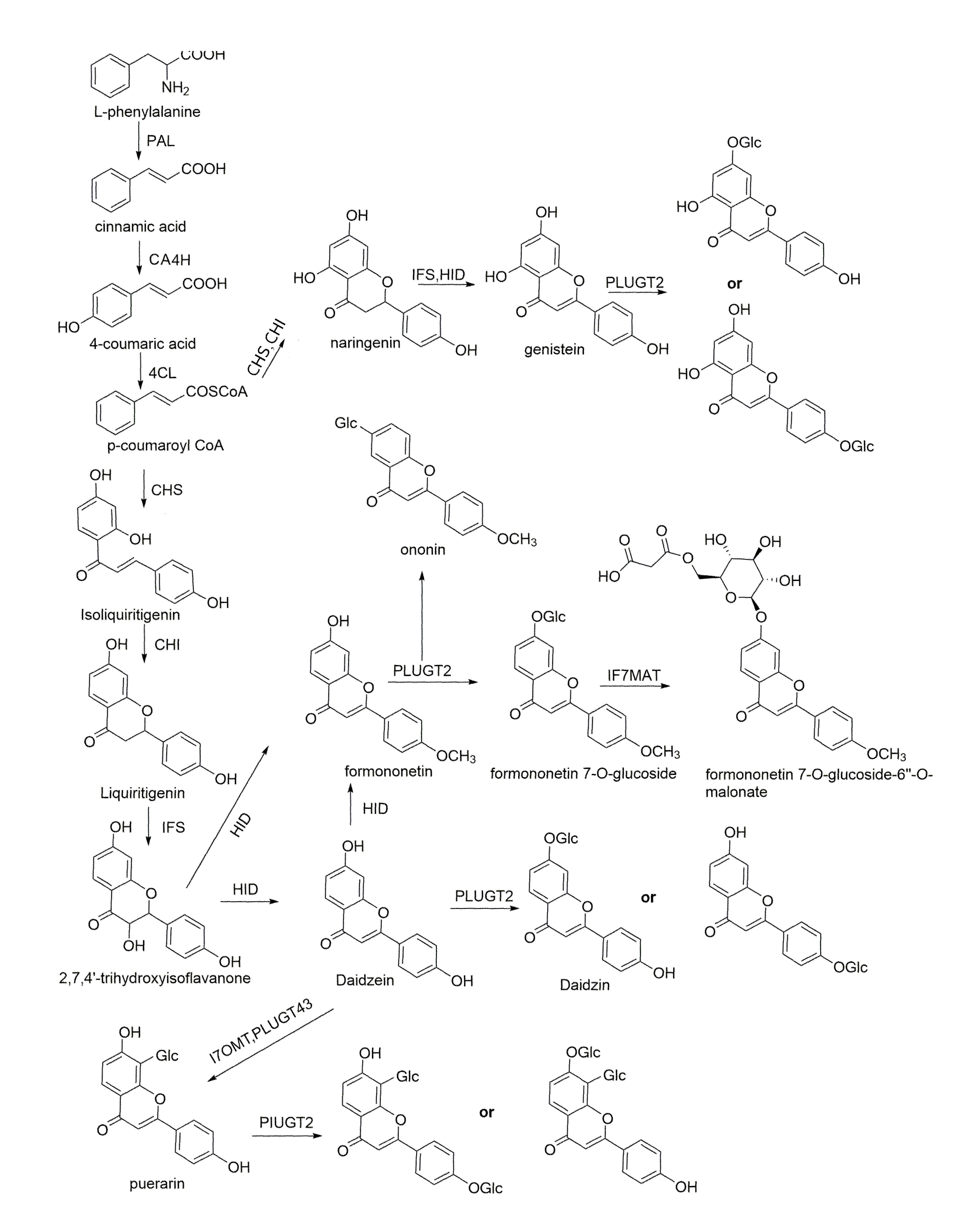

Supplement: S1 Fig — PAL: Phenylalanine Ammonia-lyase; CA4H: Trans-cinnamate4-monooxygenase; 4CL: 4-Coumarone Coenzyme A Ligase; CHS: 6-deoxychalcone synthase; CHI: Chalcone isomerase; IFS: 2-hydroxyisoflavanone synthase; HID: 2-hydroxyisoflavone dehydrates; PLUGT43: Pueraria UDP glucosyltransferase 43; PLUGT2: Pueraria UDP glucosyltransferase 2; I7OMT: isoflavone-7-O-methyltransferase. IF7MAT: isoflavone 7-O-glucoside-6''-O-malonyltransferase. (PNG) [file pone.0217593.s001.png]

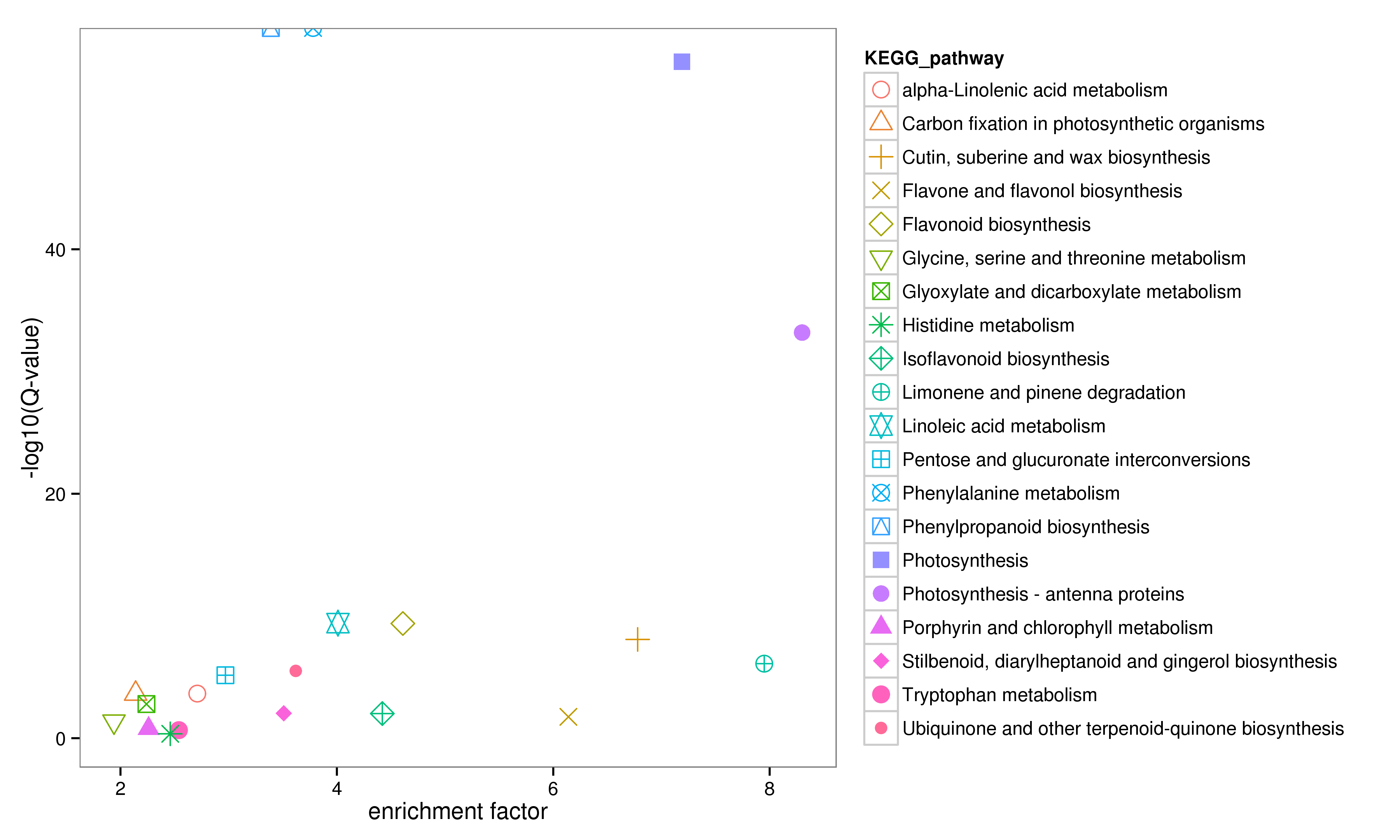

Supplement: S2 Fig — The X-axis represents the enrichment factor and log10 (Q-value). The Y-axis indicates the different KEGG pathways. (PNG) [file pone.0217593.s002.png]
