# Supplementary material for: Comprehensive transcriptome analysis reveals genes potentially involved in isoflavone biosynthesis in Pueraria thomsonii Benth
Source: PLoS One. 2019 Jun 4;14(6):e0217593. doi: 10.1371/journal.pone.0217593 (PMC6548387; doi:10.1371/journal.pone.0217593)
Supplement: S1 Table — (DOCX) [file pone.0217593.s003.docx]

**S1 Table. Primers used for qRT-PCR.**

| **Transcript name** | **Forward Primer** | **Reverse Primer** |
| --- | --- | --- |
| PB16218 | ACACAACTTGCTCCTCCGTGCT | TCTGCTCGTGCTCATTCACAGG |
| PB13397 | AACGATGTTGCTGGAACTTGCAC | TGACCAATGAAGGGAAGACGAGG |
| PB10444 | GGCACTGGGTTCCTTGGTTCA | GCACCAGGTAGGTTTGTGAGG |
| PB16388 | CCTCGCCAAAGCCTCAAACCTCA | CGGAGGCACCAGAAGTGAAGTAG |
| PB14269 | TCATCTCCTACACCACCGGAAACT | GCTTGTTCGTGGGAAATGGTTAAGT |
| B19045 | TCTAAAGCAGTGGCAATGGGACTC | TCCGTTCCTCATAGCTTGCACTG |
| PB31559 | CGTGCAGATGAAATTGCCAAGTCT | GGCGTCGTCAAGGGAATATTTATC |
| PB31589 | GCCAAGAGGCCTAAGGAGAAAGC | GATGGGTTGGCTCTCAATGTCTTG |
| PB8162（40S） | TGAAGCAGTAACTCGCAAGACTC | TGAAAGGAGCAGCATCAACCTGA |
