# Supplementary material for: Comprehensive transcriptome analysis reveals genes potentially involved in isoflavone biosynthesis in Pueraria thomsonii Benth
Source: PLoS One. 2019 Jun 4;14(6):e0217593. doi: 10.1371/journal.pone.0217593 (PMC6548387; doi:10.1371/journal.pone.0217593)
Supplement: S2 Table — (DOC) [file pone.0217593.s004.doc]

**S2 Table. PacBio data analysis.**

| **cDNA size of libraries** | **SMRT Cells number** | **Polymerase Reads** | **Post-Filter Polymerase Reads** | **Post-Filter total Number of Subread Bases** | **Post-Filter Number of Subread** | **Post-Filter Subreads N50 length** | **Post-Filter Mean Subread length** |
| --- | --- | --- | --- | --- | --- | --- | --- |
| 1-3K | 2 | 300,584 | 207,253 | 51,375,24,572 | 3,571,600 | 1,517 | 1,438 |
| 3-6K | 2 | 300,584 | 237,356 | 587,567,991 | 1,964,588 | 3,577 | 2,990 |

Polymerase reads: the number of polymerase reads sequences after sequencing; Post-filter polymerase reads: the number of polymerase reads sequences after filtration; Post-filter total number of subread bases: the number of subreads bases after filtration; Post-filter number of subread: the number of subreads after filtration; Post-filter subreads N50: subread N50 length after filtration; Post-filtermean subread length: average length of subreads after filtration.
