# Supplementary material for: Comprehensive transcriptome analysis reveals genes potentially involved in isoflavone biosynthesis in Pueraria thomsonii Benth
Source: PLoS One. 2019 Jun 4;14(6):e0217593. doi: 10.1371/journal.pone.0217593 (PMC6548387; doi:10.1371/journal.pone.0217593)
Supplement: S3 Table — (DOC) [file pone.0217593.s005.doc]

**S3** Table. Reads of insert (ROI).

| **cDNA size** | **Reads of Insert** | **Read Bases of Insert** | **Mean Read Length of Insert** | **Mean Read Quality of Insert** | **Mean Number of Passes** |
| --- | --- | --- | --- | --- | --- |
| 1-3K | 160,327 | 263,931,364 | 1,646 | 0.94 | 18.00 |
| 3-6K | 147,869 | 483,624,504 | 3,270 | 0.92 | 9.00 |

cDNA size: insert fragment size of cDNA libraries; Reads of insert: the number of ROI sequences; Read bases of insert: the total number of ROI bases; Mean read length of insert: average length of ROI; Mean read quality of insert: Quality value of ROI sequence; Mean number of passes: the mean sequencing depth of sequences in zero-mode wave.
