# Supplementary material for: Comprehensive transcriptome analysis reveals genes potentially involved in isoflavone biosynthesis in Pueraria thomsonii Benth
Source: PLoS One. 2019 Jun 4;14(6):e0217593. doi: 10.1371/journal.pone.0217593 (PMC6548387; doi:10.1371/journal.pone.0217593)
Supplement: S4 Table — (DOCX) [file pone.0217593.s006.docx]

**S4 Table. Statistics of full-length cDNA sequences.**

| **cDNA Size** | **Reads of insert** | **Number of 5’ prime reads** | **Number of 3’ prime reads** | **Number of poly-A reads** | **Number of filtered short reads** | **Number of non-full-length reads** | **Number of full-length reads** | **Number of full-length non-chimeric reads** | **Average full-length non-chimeric read length** | **Full-Length Percentage (%)** | **Artificial Concatemers（%）** |
| --- | --- | --- | --- | --- | --- | --- | --- | --- | --- | --- | --- |
| 1-3K | 160,327 | 106,920 | 114,827 | 111,762 | 18,385 | 49,253 | 92,689 | 91,385 | 1,276 | 57.81% | 1.41% |
| 3-6K | 147,869 | 85,171 | 89,051 | 87,166 | 8,745 | 71,498 | 67,626 | 67,473 | 3,174 | 45.73% | 0.23% |
| All | 308,196 | 192,091 | 203,878 | 198,928 | 27,130 | 120,751 | 160,315 | 158,858 | 2,082 | 52.02% | 0.91% |

insert fragment size of cDNA libraries; reads of insert: the number of reads of insert (ROI) sequences; Number of five prime reads: the number of ROI sequences containing 5’ primer; Number of three prime reads: the number of ROI sequences containing 3’ primer; Number of poly-A reads: the number of ROI sequences containing poly-A; Number of filtered short reads: the number of filtered ROI of <300 bp; Number of non-full-length reads: the number of non-full-length ROI; Number of full-length non-chimeric reads: the number of full-length non-chimeric ROI; Average full-length non-chimeric read length: average length of full-length non-chimeric sequence; Full-length percentage (%): the percentage of full-length sequence in ROI sequence; Artificial concatemers (%): the percentage of full length chimeric sequence in full-length sequence.
