# Supplementary material for: Comprehensive transcriptome analysis reveals genes potentially involved in isoflavone biosynthesis in Pueraria thomsonii Benth
Source: PLoS One. 2019 Jun 4;14(6):e0217593. doi: 10.1371/journal.pone.0217593 (PMC6548387; doi:10.1371/journal.pone.0217593)
Supplement: S5 Table — (DOC) [file pone.0217593.s007.doc]

**S5 Table. Statistics of Illumina-sequencing data**.

| **Sample name** | **ReadSum** | **BaseSum** | **GC(%)** | **Q30(%)** |
| --- | --- | --- | --- | --- |
| L-1 | 23,147,850 | 6,918,423,818 | 44.86 | 93.51 |
| L-2 | 23,885,655 | 7,141,775,428 | 45.13 | 93.44 |
| L-3 | 22,604,617 | 6,759,502,294 | 44.69 | 93.37 |
| S-1 | 40,145,343 | 12,001,415,820 | 44.69 | 92.90 |
| S-2 | 41,575,513 | 12,433,361,602 | 44.70 | 92.73 |
| S-3 | 34,787,110 | 10,363,469,888 | 45.08 | 93.47 |
| R-1 | 25,765,267 | 7,699,041,896 | 44.50 | 93.26 |
| R-2 | 44,547,852 | 13,312,395,168 | 44.45 | 94.07 |
| R-3 | 27,659,075 | 8,262,476,090 | 44.24 | 93.33 |

L, S and R L, S and R represent the leave, stem and root, respectively. ReadSum: Total pair-end Reads of clean data；BaseSum: Total base number of clean data：Clean data；GC(%): percentage of G and C in the clean data；Q30(%)：The base quality more than 30 in the clean data. L: leaf; S: stem; R: root.
