# Supplementary material for: Comprehensive transcriptome analysis reveals genes potentially involved in isoflavone biosynthesis in Pueraria thomsonii Benth
Source: PLoS One. 2019 Jun 4;14(6):e0217593. doi: 10.1371/journal.pone.0217593 (PMC6548387; doi:10.1371/journal.pone.0217593)
Supplement: S6 Table — (DOC) [file pone.0217593.s008.doc]

**S6 Table. Statistics of Illumina-sequencing mapping reads.**

| **Sample name** | **Total Reads** | **Mapped Reads(%)** | **Uniq mapped Reads(%)** | **Multi mapped Reads(%)** |
| --- | --- | --- | --- | --- |
| L-1 | 23,147,850 | 19,159,248 (82.77%) | 5,851,590 (30.54%) | 13,307,658 (69.46%) |
| L-2 | 23,885,655 | 20,059,482 (83.98%) | 6,093,876 (30.38%) | 13,965,606 (69.62%) |
| L -3 | 22,604,617 | 18,505,782 (81.87%) | 5,685,537 (30.72%) | 12,820,245 (69.28%) |
| S -1 | 40,145,343 | 32,710,460 (81.48%) | 10,564,510 (32.30%) | 22,145,950 (67.70%) |
| S-2 | 41,575,513 | 34,113,891 (82.05%) | 11,116,133 (32.59%) | 22,997,758 (67.41%) |
| S-3 | 34,787,110 | 28,484,551 (81.88%) | 8,952,954 (31.43%) | 19,531,597 (68.57%) |
| R-1 | 25,765,267 | 21,084,204 (81.83%) | 6,299,417 (29.88%) | 14,784,787 (70.12%) |
| R-2 | 44,547,852 | 36,457,194 (81.84%) | 11,287,011 (30.96%) | 25,170,183 (69.04%) |
| R-3 | 27,659,075 | 21,937,024 (79.31%) | 6,877,843 (31.35%) | 15,059,181 (68.65%) |

L, S and R represent leave, stem and root, respectively.
