# Supplementary material for: Comprehensive transcriptome analysis reveals genes potentially involved in isoflavone biosynthesis in Pueraria thomsonii Benth
Source: PLoS One. 2019 Jun 4;14(6):e0217593. doi: 10.1371/journal.pone.0217593 (PMC6548387; doi:10.1371/journal.pone.0217593)
Supplement: S7 Table — (DOC) [file pone.0217593.s009.doc]

**S7 Table.** Functional annotation based on public databases.

| **Database** | **Annotated Number** | **300 ≤ length（bp） <1000** | **Length（bp）≥1000** |
| --- | --- | --- | --- |
| Nr | 43,083 | 5,549 | 37,534 |
| Swissprot | 32,079 | 3,423 | 28,656 |
| KEGG | 17,391 | 2,039 | 15,352 |
| GO | 24,720 | 3,104 | 21,616 |
| COG | 17,185 | 1,607 | 15,578 |
| eggNOG | 40,870 | 4,982 | 35,888 |
| KOG | 25,626 | 2,633 | 22,993 |
| Pfam | 35,525 | 3,740 | 31,785 |
| All Annotated | 43,195 | 5,584 | 37,611 |
